# Rickettsial Infections Are Neglected Causes of Acute Febrile Illness in Teluk Intan, Peninsular Malaysia

**DOI:** 10.3390/tropicalmed7050077

**Published:** 2022-05-18

**Authors:** Muhamad Yazli Yuhana, Borimas Hanboonkunupakarn, Ampai Tanganuchitcharnchai, Pimpan Sujariyakul, Piengchan Sonthayanon, Kesinee Chotivanich, Sasithon Pukrittayakamee, Stuart D. Blacksell, Daniel H. Paris

**Affiliations:** 1 Department of Clinical Tropical Medicine, Faculty of Tropical Medicine, Mahidol University, Bangkok 10400, Thailand; aleeyuhana@hotmail.com (M.Y.Y.); nok@tropmedres.ac (K.C.); yon@tropmedres.ac (S.P.); 2 Department of Infectious Diseases and Tropical Medicine, School of Medicine, Universiti Teknologi MARA (UiTM), Sg Buloh Campus, Sungai Buloh 40600, Selangor, Malaysia; 3 Mahidol-Oxford Tropical Medicine Research Unit, Faculty of Tropical Medicine, Mahidol University, Bangkok 10400, Thailand; ampai@tropmedres.ac (A.T.); ps.pimpan@hotmail.com (P.S.); stuart@tropmedres.ac (S.D.B.); 4 Department of Molecular Tropical Medicine and Genetics, Faculty of Tropical Medicine, Mahidol University, Bangkok 10400, Thailand; piengchan@tropmedres.ac; 5 Centre for Tropical Medicine, Nuffield Department of Clinical Medicine, Churchill Hospital, Oxford OX3 7FZ, UK; 6 Faculty of Medicine, University of Basel, 4003 Basel, Switzerland; daniel.paris@unibas.ch; 7 Department of Medicine, Swiss Tropical and Public Health Institute, 4123 Allschwil, Switzerland

**Keywords:** rickettsial, Malaysia, acute febrile illness

## Abstract

Rickettsial infections are among the leading etiologies of acute febrile illness in Southeast Asia. However, recent data from Malaysia are limited. This prospective study was conducted in Teluk Intan, Peninsular Malaysia, during January to December 2016. We recruited 309 hospitalized adult patients with acute febrile illness. Clinical and biochemistry data were obtained, and patients were stratified into mild and severe infections based on the sepsis-related organ failure (qSOFA) scoring system. Diagnostic assays including blood cultures, real-time PCR, and serology (IFA and MAT) were performed. In this study, pathogens were identified in 214 (69%) patients, of which 199 (93%) patients had a single etiology, and 15 (5%) patients had >1 etiologies. The top three causes of febrile illness requiring hospitalization in this Malaysian study were leptospirosis (68 (32%)), dengue (58 (27%)), and rickettsioses (42 (19%)). Fifty-five (18%) patients presented with severe disease with a qSOFA score of ≥2. Mortality was documented in 38 (12%) patients, with the highest seen in leptospirosis (16 (42%)) followed by rickettsiosis (4 (11%)). While the significance of leptospirosis and dengue are recognized, the impact of rickettsial infections in Peninsular Malaysia remains under appreciated. Management guidelines for in-patient care with acute febrile illness in Peninsular Malaysia are needed.

## 1. Introduction

The major treatable and preventable causes for acute febrile illness (AFI) in Southeast Asia are increasingly being recognized [1,2,3,4,5,6,7,8]. Dengue and malaria are amongst the most reported etiologies, but rickettsioses—although identified in multiple studies—remain underappreciated despite their nature of being easily treatable and extent of disease burden [9,10]. Coined as neglected tropical diseases, the tropical rickettsial illnesses consist of the chigger-borne infection known as scrub typhus caused by *Orientia tsutsugamushi*, the flea-borne murine typhus by *Rickettsia typhi*, and the tick-borne spotted fevers due to numerous *Rickettsia* spp. [9]. Recent studies in Thailand, Laos, and Bangladesh, bordering with Myanmar, have shown that scrub typhus and other rickettsial infections are among the leading causes of fever in the local communities [2,3,4,8]. Malaysia is divided geographically into the more urbanized Peninsular Malaysia and more rural Malaysian Borneo, with differing regional endemic disease profiles. For example, malaria is prevalent in Borneo [11,12,13,14], while leptospirosis and dengue are more commonly reported in rural and urban areas of Peninsular Malaysia, respectively [15,16,17,18,19,20,21,22]. Although a strong emphasis on scrub typhus was seen in Malaysian history dating back to the early 1900s (then known as the Federated Malay States), when Dowden first described a 26-year-old plantation worker in Kuala Lumpur with what he described as “*kedani fever*” (*kedani* is Japanese for “hairy mite”) [23]. In 1924, scrub typhus was first distinguished from murine typhus through a mistake in Proteus strains used for serological diagnosis [24] by Fletcher from the Institute for Medical Research (IMR) in Kuala Lumpur, who described the first largest case series of scrub typhus amongst the local plantations workers [25]. During World War II, the IMR represented the epicenter for scrub typhus studies assisting the Allied troops; these involved the evaluation of a scrub typhus vaccine, characterization of mites and associated disease transmission, as well as pursuing studies in the treatment and prevention of scrub typhus [26,27,28,29,30]. By the time the British left the country in 1957, and chloramphenicol was found as effective treatment of scrub typhus, all interest in rickettsiology waned, and the number of studies conducted has since dwindled. However, two decades later, in the 1970s, scrub typhus was still a predominant disease according to an epidemiological study conducted by the IMR [31]. In the modern era, Tay ST et al. from the IMR continued this legacy and spearheaded the diagnostic advances in tropical rickettsial illnesses amongst the local communities; notably, the sero-prevalence findings were as high as 33% in one of her study populations [32,33,34]. Since then, no comprehensive study incorporating both epidemiological and clinical aspects using updated diagnostic methodologies on rickettsia is available. Thus, we conducted a prospective study looking for the causes of acute febrile illness (AFI), including the rickettsial diseases (scrub typhus, murine typhus, and spotted fever rickettsioses), in Peninsular Malaysia.

## 2. Material and Methods

### 2.1. Ethics Statement

Full ethical clearance was obtained from the Malaysian National Medical Research Register Ethical Committee (NMRR-15-756-25320) and the Ethic Committee of the Faculty of Tropical Medicine, Mahidol University (MUTM 2016-028-01). All patients enrolled provided written informed consent prior to collection of sample specimens.

### 2.2. Study Design and Patient Selection Criteria

**Figure 1:**
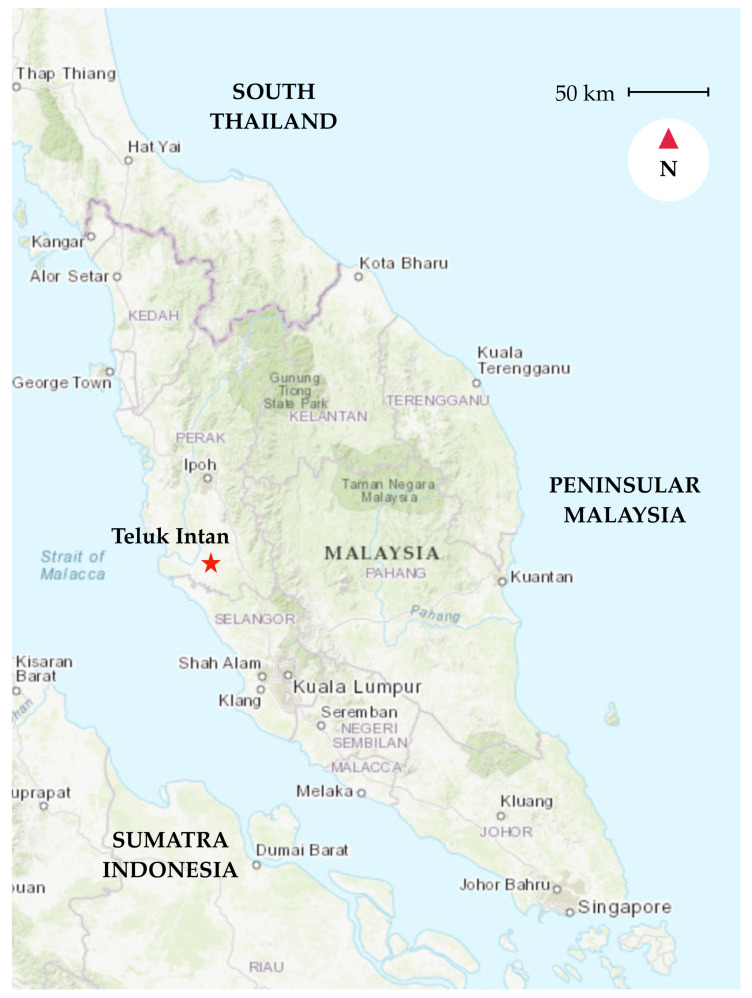
Map of Peninsular Malaysia showing the location of Teluk Intan (red star) within the State of Perak. Accessed on 13 May 2019 from https://landsatlook.usgs.gov/viewer.html.

We conducted this prospective study for a full calendar year from 1 January until 31 December 2016 in Teluk Intan Hospital, a tertiary hospital located in the rural part of midwest Peninsular Malaysia (Figure 1). The hospital is equipped with 300 medical beds, including an infectious diseases ward. We included hospitalized patients aged >18 years old complaining of fever defined as tympanic temperature of >38 °C that lasted for <14 days. All patients were enrolled upon hospital admission and followed up until hospital discharge or death. Nosocomial infections (defined as fever with history of hospitalization in previous 90 days) were excluded. The Quick Sequential Organ Failure Assessment (qSOFA) scoring system was used to stratify for disease severity [35].

### 2.3. Patient Data, Samples, and Biochemistry/Biomarkers

Demographic, clinical, and laboratory data of patients were individually collated on study case-record forms (CRFs) from hospital records. A total of 17 mL blood was collected upon enrollment (9 mL whole blood into EDTA and plain tube for blood analysis and diagnostics, 8 mL for blood culture) and 3 mL upon discharge. Blood was collected upon enrollment for full blood count (Sysmex XN-1000-Hematology Analyzer, Sysmex Singapore Pte Ltd., Singapore) and renal and liver biochemistry (Beckman Coulter AU680-Chemistry Analyzer, Beckman Coulter, CA, USA). Assessed biomarkers included C-reactive proteins (CRP) (Immune Quantitative Analyzer) and procalcitonin (PCT) (Procalcitonin Rapid Test Kit, Easy Diagnosis Biomedicine Co., Ltd., Wuhan, China).

### 2.4. Diagnostic Laboratory Assays Performed

*Nucleic acid analyses*. A total of 4 mL of whole blood was obtained in EDTA upon enrollment for molecular analysis. Nucleic acids were extracted using the QIAamp DNA minikit (QIAGEN, Valencia, CA, USA), and real-time PCRs were run in triplicate on a Bio-Rad machine (CFX96 Real-Time PCR system). PCR analyses were conducted to diagnose leptospirosis and rickettsioses. A convalescent sample of 3 mL of whole bloods were collected in plain tube before patient discharge.

*Blood cultures*. A total of 8 mL of whole blood were drawn into an aerobic bottle and were immediately incubated using the BACTEC 9240 system up to 5 days in the hospital microbiology laboratory (Becton Deckinson, Franklin Lakes, NJ, USA) [36]. Positive samples were plated in Columbia sheep blood, chocolate, and MacConkey’s agars, and identification was carried out using the conventional biochemical methods [2]. Coagulase-negative *Staphylococcus aureus* and *Bacillus* spp. were regarded as culture contaminants.

*Dengue infection.* The dengue virus ELISA (Pan Bio, Brisbane, Australia) was used to detect dengue NS1, anti-dengue IgM, and IgG antibodies from patient sera. Using the IgM to the IgG ratio, we defined primary dengue as a ratio of ≥1.8 and secondary dengue when the ratio was <1.8 [37]. We did not perform dengue PCR or serotype analysis in this study.

*Rickettsial infections.* The diagnosis of scrub typhus and murine typhus was based on both real-time PCR (qPCR) and immunofluorescence assays (IFA), and spotted fever rickettsioses were diagnosed by qPCR only. Scrub typhus PCR assays targeted the 47 kDa gene (qPCR) and the 56 kDa gene (nested conventional PCR). The assays, primers, and probe used were previously described [38,39]. PCR for murine typhus and spotted fever rickettsioses involved a first qPCR based on the 17 kDa gene targeting the *Rickettsia* genus and a second nested PCR to detect the *ompB* gene for murine typhus. The assays, primers, and probe sequences were previously described [3,8]. Diagnosis of murine typhus by PCR was made if both 17 kDa and *ompB* genes were detected. Diagnosis of spotted fever rickettsial typhus was made on positive 17 kDa real-time PCR and negative *ompB* gene [40,41]. Serological diagnosis detected IgM antibodies against *O. tsutsugamushi* and *R. typhi* by IFA (Australian Rickettsial Reference Laboratory, Geelong, Australia) [42]. The positivity cutoff was an IgM titer of 1:400 or more or if paired sera showed a four-fold rise [43,44]. 

*Leptospirosis*. Leptospiral infection was diagnosed by either PCR or serologically by microagglutination tests (MAT). We based our molecular detection of *Leptospira* spp. using the *Leptospira* lipL32 and 16S rRNA genes [45]. The MAT panel consisted of 17 serovars; 3 locals (IMR LEP 1, IMR LEP 115, and IMR LEP 175) and 14 WHO-proposed serovars (Australis, Autumnalis, Batavia, Canicola, Celledoni, Grippotyphosa, Hardjoprajitno, Icterohaemorrhagiae, Javanica, Pyrogenes, Tarrasovi, Djasiman, Patoc, and Pomona). A positive MAT result was defined as a serum titer of 1:400 or more or if paired serum showed a four-fold rise [22,46,47]. 

*Malaria, pulmonary tuberculosis, and HIV*. Malaria was diagnosed with thin and thick blood smears (performed in duplicates), *M. tuberculosis* by the acid-fast bacilli stain on three expectorated sputum samples, and HIV using the 4th-generation p24 antigen and antibodies detection test [48]. 

Blood culture was not performed for fungal pathogens or tuberculosis, and diagnostics did not include influenza, seasonal coronaviruses, Japanese encephalitis, chikungunya, and the viral hepatitis.

### 2.5. Attribution to Final Diagnosis

The final diagnosis was attributed to each case by the strength of evidence supporting each diagnosis: Strongest diagnostic evidence in decreasing order was: (I) PCR/antigen/culture positivity > (II) dynamic serology (4-fold rise) > (III) single titer and/or serological positivity cut-off titer > (IV) visualized pathogens by microscopy [4]. In this study, the final diagnosis was made using results from culture (blood culture); antigen detection (dengue NS1, HIV p24); and nucleic acid detection by PCR (*Orientia tsutsugamushi*, *Rickettsia typhi*, *Rickettsia* spp., and *Leptospira* spp.); serology (*O. tsutsugamushi* and *R. typhi* IFA, leptospirosis MAT); and blood films for malaria and the AFB stain for mycobacteria (*M. tuberculosis*). 

### 2.6. Statistical Analysis

We analyzed the association between patient symptoms, signs, and laboratory features for each etiological diagnosis using STATA, (Stata/MP 14.1 for Mac (64-bit Intel) StataCorp 4905 Lakeway Dr, College Station, TX 77845, USA). Variables were described using median and interquartile range (IQR) for continuous data and frequencies and percentage for categorical data. Wilcoxon rank-sum test was used for continuous data analysis, whereas chi-square and Fisher’s exact testing were used for categorical data. 

## 3. Results

**Figure 2:**
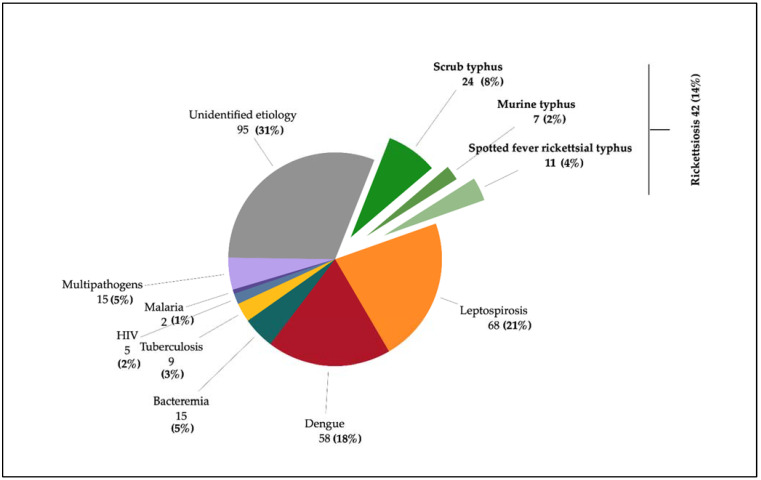
The etiologies of acute febrile illness in Teluk Intan from January 2016 until December 2016.

**General findings.** In this study, an etiology (i.e., an identified pathogen) could be attributed to 214/309 patients (69%) (Figure 2). Out of this group, 199/214 patients (93%) had single pathogen infection, and 15 (5%) patients had at least two or more pathogens diagnosed. When only single diagnoses were considered, 68/214 (32%) had leptospirosis, 58 (27%) dengue, and 42 (19%) rickettsial infections (of which 24 (11%) scrub typhus, 11 (5%) spotted fever rickettsioses and 7 (3%) murine typhus). Only 15 patients (7%) presented with documented bacteremia, and the top three blood culture results were *Staphylococcus aureus, Klebsiella pneumoniae,* and *Burkholderia pseudomallei*. Nine (4%) had tuberculosis, five (2%) had newly diagnosed HIV, and two (1%) malaria, with one case of *Plasmodium knowlesi* and *P. malariae* each. Out of all of the 15 patients with co-infections, none was diagnosed with more than one type of rickettsial infections. 

**Demographic, clinical, and diagnostic findings**. A total of 358 patients were screened for recruitment into the study over the 12-month period in which 313 (87%) patients met the inclusion criteria. Of these, 309 (98%) patients consented and had complete clinical and admission biochemistry data available. Convalescent sera were available in 243 patients (78%) with a median interval between paired samples of 5 days (IQR 3–6 days). Gender distribution was similar with males (171, 55%) and females (138, 45%) represented in this cohort. (*p* = 0.06). The median age was 47 years old (IQR 29–62 years old). Malay ethnicity predominated with 194 (63%), followed by Indian at 84 (15%), Chinese at 30 (11%), and Aborigine at 30 (10%). 161 patients (52%) participated in agricultural activities. 107 patients (35%) had at least one co-morbidity with diabetes documented most commonly in 83 patients (27%). 46 (50%) patients received antibiotics prior to hospitalization. The median duration of fever was 4 days (IQR 2–7). Fever aside, the top five reported symptoms were cough (50%), vomiting (40%), headache (35%), diarrhea (35%), and myalgia (27%). The commonest clinical signs elicited were abnormal lung sounds (30%), rash (8%), and lymphadenopathy (5%). Eschar was found in only three patients (0.9%) (Table 1). 

The median hospital stay was 6 days (IQR 4–9). Mortality for the entire cohort during the hospital stay was 38/309 (12.3%) of all patients. Those who presented with severe illness (qSOFA score ≥2) were significantly associated with subsequent death (OR 54.6 (20-8-143.2; 95% CI), *p* < 0.001). Amongst those patients with a final diagnosis (n = 214, 69%), those diagnosed with leptospirosis had the highest mortality with 16/38 (42%) deaths, followed by rickettsiosis with 4/36 (11%) deaths (1 scrub typhus and 3 spotted fever rickettsial typhus infections).

**General laboratory findings.** The median white cell count was 8.2 × 10^9^/L (IQR 4.4–14.6). Leucocytosis was found in 109 patients (34%) and leukopenia in 71 patients (23%). The median platelet count was 179 × 10^9^/L (IQR 91–283), and 132 patients (43%) had thrombocytopenia. The median serum creatinine level was 79 umol/L (IQR 61–120). Acute kidney injury was diagnosed in 82 (26%). The median CRP level was 68.7 mg/L (IQR 13–153 mg/L) and procalcitonin (PCT) of 0.5 ng/dL (0.17–7.6 ng/dL). Around half of the patients (150, 50%) had raised PCT plasma levels of >0.5 ng/dL. Using the qSOFA score, 254 (82%) presented with mild disease. Overall, 185 (60%) scored of 0; 69 (22%) had a score of 1; 55 (18%) patients had a score of severe disease; 40 (13%) patients had a score of 2; and 15 (5%) had score of 3. 

**Scrub typhus.** The diagnosis of scrub typhus was made in 24/214 patients (11%) cases, which was confirmed by both PCR and IFA in two patients, by PCR alone in twenty patients, and by IFA alone in two patients. Of the four positive cases involving a positive IFA result, three patients had demonstrated at least a four-fold rise in the convalescent IFA titers. Two patients had admission IFA titers of 1:400, respectively, and two patients of 1:12,800, with one of them demonstrating a dynamic rise in titer from 1:12,800 to 1:25,600 in the convalescent sera. Most patients were male (14, 58%), and interestingly, six (25%) came from the minority Aborigine ethnicity, which equaled to 20% (6/30) of this ethnic group in this study. Majority of the patients (50, 60%) lived in the rural area. The median fever days prior to admission was 5 days (IQR 2–7), and the median hospitalized days was 7 days (IQR 6–10.5). The top five most common complaints were cough (46%), headache (42%), vomiting (42%), diarrhea (38%), and arthralgia (25%). Only one patient had an eschar identified. The median platelet counts were 182 × 10^9^/L (IQR 110–236) and white blood cell counts of 8.5 × 10^9^/L (IQR 6.1 × 14.6). The median creatinine level was 76 umol/L (IQR 62.5–110). The median CRP level on admission was 49.1 mg/dL (IQR 8.6–84.3) and procalcitonin of 0.38 ng/mL (IQR 0.12–1.2). Twenty patients had mild infections, and four patients had severe infections by the qSOFA score, and out of this, one patient died. When biomarkers were analyzed between the “mild” and “severe” disease severity groups, both CRP and PCT plasma levels were higher in the severe group, but only PCT value reached statistical significance: median CRP level of 103.0 ng/dL (IQR 59.1–166.6) versus 39.0 mg/dL (IQR 7.3–84.3) (*p* = 0.27) and PCT level of 3.2 ng/mL (0.63–10.0) versus 0.33 mg/mL (0.12–0.86) ng/dL (*p* = 0.02), respectively.

**Murine typhus.** A total of seven patients were diagnosed with murine typhus, in which six patients were diagnosed by PCR alone and one patient by IFA alone. In the one case by IFA alone, the 39-year-old man had admission titers of 1:3200, which increased to 1:12,800 on the repeated sera 5 days apart. No patients had both PCR and IFA detected. The overall median age was 31-year-old (23–70), and median fever duration was 3 days (IQR 2–10). Six (90%) live in urban areas, and no seasonality trend was seen. The most frequent symptoms reported were myalgia (57%), arthralgia (43%), cough (43%), diarrhea (43%), and vomiting (29%). Only one patient had a skin rash. The median PCT level was 0.45 ng/mL (IQR 0.13–0.98), which is almost similar with cases of scrub typhus: 0.4 ng/mL (IQR 0.5–1.2). All the murine cases were mild, with no death documented. 

**Spotted fever rickettsial typhus.** This was diagnosed in 11 patients by PCR alone. The median age was 43 years old (IQR 26–53). The median fever day was 3 days (IQR 2–7). The commonest symptom was myalgia (53%) followed by arthralgia, headache, diarrhea, and vomiting,, 45% each respectively. There were two cases with eschar identified. Seven patients had mild disease, and four patients had severe disease, in which three patients died, each with clinical suspicions of central nervous system infections. 

**Leptospirosis**. A total of 68 (32%) of 214 patients were diagnosed with leptospirosis, in which 10 patients had both positive-identified PCR and MAT, 36 had positive PCR alone, and 22 had positive MAT alone. Of the 32 MAT, the identified serovars were local IMR Lepto 1, IMR Lepto 175 Autumnalis, and Patoc serovar. Based on the phylogenetics analysis, *Leptospira interrogans* was the leading cause in 26 patients, followed by *L. kirschneri* in two patients and one patient with *L. kmetyi.* The median age was 54 years (IQR 32–64). The median fever days was 4 days (IQR 2–7). Interestingly, only 22 (33%) patients had severe infection, with total of 16 (22%) deaths documented, which was the highest amongst those with a microbiological diagnosis.

**Dengue**. The most common infection diagnosed in this cohort was dengue fever, with 63 patients (29%) in total, of which 58 had mono-infection, and 5 had co-infections. Dengue was the second commonest diagnosis and the most prevalent viral illness seen in this cohort. Fifty-eight patients (20%) had dengue infection diagnosed by positive NS1 ELISA antigen alone. The median age was 28.5 years old (IQR 22–47). When ratios of IgM to IgG were analyzed, almost equal numbers were seen between primary and secondary dengue infection: 30 patients (47%) and 33 patients (52%), respectively. There were no dengue deaths documented throughout the study period. 

**Comparison between leptospirosis, dengue, and all rickettsial infections.** When we compare between the three most prevalent diseases, there were no significant associations seen between the demographic data or clinical signs/symptoms associated with the diagnosis (Table 2). However, reduced procalcitonin and CRP were significantly associated with dengue infection compared to leptospirosis and rickettsiosis. 

**Figure 3:**
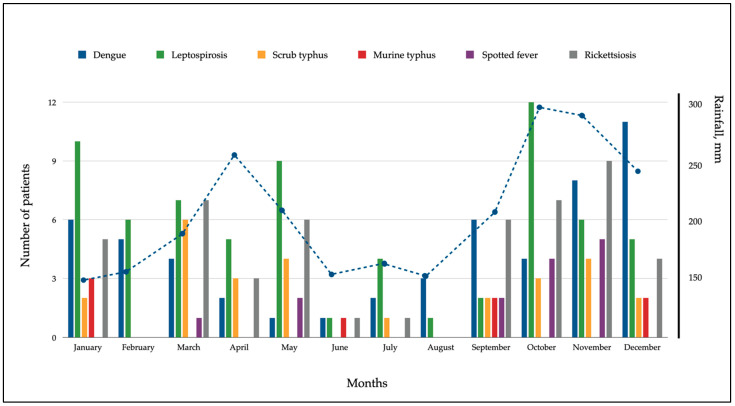
Seasonality of causes of AFI in patients admitted to Teluk Intan Hospital, Malaysia, from January 2016 until December 2016. The dashed line represents monthly rainfall in millimeters. Source: the Malaysia Meteorological Department, Teluk Intan.

**Seasonality.** A distinct biphasic pattern of scrub typhus admissions to Teluk Intan Hospital was observed during the study period, coinciding with the wet and rainy seasons (Figure 3). The first peak was seen during the first rainy season in March and May followed by with very few cases seen in the dry month between June and August. The second peak occurred in the subsequent rainy season from September through December in the final quarter of the study period. Similar to scrub typhus, a high number of dengue cases were seen during the rainy seasons particularly in the later part of the year. A consistent number of leptospirosis, murine typhus, and spotted fever rickettsial typhus cases were seen throughout the study period without marked association to the wet seasons.

**Antimicrobial treatment findings.** A total of 238 (75%) patients received either empirical or targeted antibiotics during the hospitalization period. The two most commonly prescribed antibiotic regimens were ceftriaxone and ampicillin-sulbactam with or without azithromycin or doxycycline. Interestingly, no patients in the rickettsial group received a monotherapy of an anti-rickettsial agent alone. Out of seven patients in the rickettsial group who did not receive any anti-rickettsial agent, four died (untreated mortality of 57%). Only nine (15%) patients in the dengue group received antibiotic(s).

## 4. Discussion 

This prospectively conducted “causes-of-febrile illness” study over one full calendar year highlights the substantial contribution of rickettsial infections to the burden of AFI in Malaysia and how underappreciated these diseases remain today [10]. With regards to rickettsial infections among hospitalized patients in Malaysia, the following findings require consideration: 

To date, scrub typhus and other rickettsial infections remain among the predominant etiologies of AFI in this part of Peninsular Malaysia, which is similar to recent findings in clinical epidemiological studies in the surrounding countries [2,3,4,5,8].

Our study incorporated the qSOFA scoring system (a universal prognostication method used for sepsis) to distinguish between mild and severe disease severity [35]. The clinical impact of rickettsioses was emphasized in that 2 of 10 patients with rickettsial infections presented with severe manifestations (qSOFA scores of ≥2), and 1 in 10 patients died. Indeed, the disease is easily treatable but only if recognized and diagnosed correctly and followed up with early and appropriate treatment. However, recognizing rickettsial infections is challenging, as predominant symptoms and clinical signs are usually non-specific. The non-specific nature of clinical patterns described in our study are consistent with the Korean and Indian populations describing the common symptoms of headache and respiratory complaints [49,50]. 

A low eschar prevalence was seen in the confirmed cases of our cohort, which is comparable to findings in areas of high scrub typhus endemicity, such as Chittagong, Bangladesh [3], and suggests that pre-existing immunity could be wide-spread in the catchment area of Teluk Intan hospital, as the formation of an inoculation lesion and eschar can be much milder or absent in pre-exposed individuals [38]. The absence of an eschar in many scrub typhus patients makes this a great mimicker of multiple etiologies of AFI.

This study highlights the added value of incorporating PCR as a diagnostic modality. Without using PCR, 85% of the scrub typhus cases and 91% of the murine typhus cases in this cohort would have been undiagnosed and missed. PCR is useful within the first 10 days of infection, and as the median “fever days” to presentation in this cohort was 5 days for rickettsial patients, this is a clinically highly relevant modality [3,38]. There is an urgent need to expand our diagnostic capacity using molecular detection assays to increase the diagnostic sensitivity and capacity of detecting bacteremia in a timely way before antibody responses become detectable. In remote areas where molecular diagnostics are not feasible, point-of-care tests will be an asset in improving the diagnosis of rickettsial infections. 

With regards to biomarkers, only a handful of studies have described the utilization of either procalcitonin (PCT) or CRP for clinical decision making in rickettsial infections [8,51,52,53,54]. Lee et al., used PCT to distinguish between scrub typhus infection and *Escherichia coli* bacteremia and found that the PCT levels were markedly higher in the later condition [51]. A similar trend was observed in this study with our median of PCT plasma levels in the bacteremia group being higher than the median of plasma levels in the scrub typhus group (4.6 ng/mL (IQR 0.37–25) versus 0.38 ng/mL (IQR 0.12–1.2), respectively (*p* < 0.001)). When comparing both CRP and PCT levels for all bacterial infections in this study (bacteremia plus leptospirosis plus rickettsiosis) against viral infections, both biomarkers were significantly higher in all bacterial groups than in the dengue group, with a median CRP plasma level of 93 mg/L (IQR 19–166) vs. 5.8 mg/L (2.8–18.1) and PCT of 0.90 ng/mL (IQR 0.23–15) vs. 0.29 ng/mL (IQR 0.15–0.65), respectively (both *p* < 0.001). Our study provides added evidence that raised admission CRP or PCT plasma levels could assist in differentiating bacterial infections from dengue infection, which would support the decision for initiation of antibiotics (or not) in both group, and PCT could be used to distinguish acute scrub typhus from the culturable bacterial infections. In addition, this study suggests that PCT and CRP could also serve as prognostic biomarkers for the outcome of a scrub typhus infection, which is in accordance to previous studies [8,51,52,54]. However, well-powered prospective studies dedicated to addressing these specific questions are necessary to clarify the utility of these biomarkers and provide positivity cutoff levels.

The high prevalence of leptospirosis was anticipated, as this zoonotic infection has been widely reported and studied in the country [55,56]. In this cohort, *L. interrogans* was the most common isolated species amongst our febrile patients. A rare and special finding was that we diagnosed a patient with *L. kmetyi* infection, which is one of very few clinical cases reported in Malaysia [57,58]. The use of both PCR and MAT for diagnosing leptospirosis in this study increased the chance of disease detection in a similar way in that early diagnosis is facilitated by direct pathogen detection in blood during the bacteremia phase [45,59,60].

Although we did not perform autopsy to determine the real causes of death in this study, the high number of deaths in the leptospirosis group were associated to the presentation with advanced disease and high disease severity; one-third of these patients presented with a qSOFA score of ≥2 upon admission, and almost one-third overall had *L. interrogans* infection, which is of high human pathogenicity and associated with increased mortality [56]. Almost half of the patients, n = 29 (40%), demonstrated impaired kidney function with raised serum creatinine levels, which may also have affected the pharmacokinetics/pharmacodynamics of the antibiotics given and may contribute to the number of deaths in this group, which is an underappreciated factor [61]. Hence, further studies on the time of antibiotics delivery and the pharmacokinetics/pharmacodynamics of the antibiotics in this group of patients coupled with disease severity dynamics would provide more insight on the disease outcomes [61].

No demographic or clinical variables differed significantly between rickettsial and leptospiral illnesses. However, in this study, the comparison of biomarkers between these infections demonstrated that the median CRP and PCT plasma levels were higher in leptospirosis than in rickettsiosis. High CRP biomarker has been described in severe leptospirosis [8,62,63]. Thus, these biomarkers may be useful in differentiating between these two common zoonotic infections when other tests are not available or inconclusive.

Notably, before the COVID-19 pandemic, dengue was one of the commonest viral infections that required hospitalization in Malaysia [21,64]. Unlike for other etiologies of AFI, an easy-to-use rapid test kit for dengue was made widely available in the country and this facilitated disease recognition by local trained physicians. Hence, it is not surprising that no deaths were recorded in the dengue group in this cohort given the higher level of awareness, earlier presentation with the median fever days of 3 days (IQR 2–5), and better national management guidelines compared to other causes of AFI [65].

The Malaysian Borneo of Sabah and Sarawak is the epicenter of malaria where severe knowlesi malaria is very common, with prevalence ranging from 9 to 29% from studies in the Borneo [11,12,13]. The two malaria cases diagnosed in our study (one *Plasmodium knowlesi* and *P. malariae* each) reflect the much smaller number of malaria cases in Peninsular Malaysia [66].

There were only about 5% of patients in our cohort with co-infection. Overall, the number of patients with fever presenting with more than one infective pathogen is not common, similar to other epidemiological studies, for example, in Bangkok (9%) or Laos (13%) [2,4].

The high number of scrub typhus cases during the rainy seasons in our study supported the seasonal distribution of scrub typhus seen in previous studies [3,8]. Interestingly, the rainy months of each country are different. Therefore, the majority of scrub typhus patients in this study were found during March–May and September–December, while the majority of patients in the study from northern Thailand were found during June to November [8]. Nonetheless, empirical treatment using doxycycline in patients presenting during the raining seasons perhaps could be included in a treatment algorithm while waiting for a confirmatory tests. The small number of murine typhus cases in our study did not reflect the seasonal distribution that was demonstrated in a previous study in Bangladesh [3].

This study has some limitations that need to be emphasized; the single-center study design limits the generalization and application of results. The high number of undiagnosed patients may be due to lack of testing towards other common respiratory viral illnesses, such as influenza and the seasonal coronaviruses, as well as the mosquito-borne chikungunya and Zika viruses. We did not succeed in obtaining paired samples (i.e., convalescent sera) in about one-third of patients in this study, which may affect the actual number of leptospirosis, scrub typhus, and murine typhus. In addition, the duration between our enrollment and convalescent samples was rather short, with a median of 5 days in which this may not be ample enough to show a significant four-fold rise.

In summary, this study highlights that apart from dengue and leptospirosis, the rickettsial infections represent an important and under-recognized cause of AFI in Peninsular Malaysia. An empirical treatment strategy using doxycycline or azithromycin may be beneficial in certain cases, but this approach requires careful evaluation. Physicians should be aware of the zoonotic bacterial infections such as leptospirosis and rickettsiosis in patients with AFI, as these two causative agents are not easily isolated/cultured in routine blood culture. Rickettsial illnesses are easily treatable but only if recognized early and diagnosed correctly. Hence, improving general awareness, knowledge, and access to molecular diagnosis is important to reduce the associated morbidity and mortality of rickettsial illnesses in Malaysia.

## Figures and Tables

**Table 1:** Demographic and clinical data of 309 enrolled patients.

Demographic and Clinical Data	Total n = 309
Median age in years (IQR)	47 (IQR 29–62)
Male sex	171 (55%)
Underlying medical co-morbidity/-ies	109 (34%)
Median fever day(s) before hospitalization (IQR)	4 (IQR 2–7)
Antibiotic consumption pre-hospitalization	46 (15%)
Ethnicity	
• Malay	194 (63%)
• Indian	48 (15%)
• Chinese	34 (11%)
• Orang Asli (Aborigine)	30 (10%)
Symptoms and signs	
• Cough	154 (50%)
• Vomiting	121 (40%)
• Headache	107 (35%)
• Diarrhea	106 (34%)
• Myalgia	83 (27%)
• Acute confusion or new-onset seizure	24 (8%)
• Bronchial breathing or reduced air entry	94 (30%)
• Rash	23 (7%)
• Lymphadenopathy	16 (5%)
• Hepatomegaly	14 (5%)
• Splenomegaly	8 (3%)
• Eschar	4 (1%)
Sepsis severity and outcome	
• Median length of hospital stays in days (IQR)	6 (4–9)
• Severe sepsis by qSOFA (≥2) ^†^	55 (18%)
• In-patient antibiotic (s)	238 (75%)
• Deaths	38 (12%)

Data are n (%) unless otherwise indicated. ^†^ Quick sequential organ failure assessment (qSOFA); 1 point each for systolic blood pressure < 100 mmHg, respiratory rate > 22/min, and altered mental status with Glasgow coma scale < 15.

**Table 2:** Demographic and clinical comparisons between all rickettsial infections, leptospirosis, and dengue.

Demographic and Clinical Data	* All Rickettsial Infections; n = 42	Leptospirosis; n = 68	Dengue; n = 58	*p*-Value
Median age in years (IQR)	43 (IQR 27–60)	56 (IQR 34–64)	29 (IQR 22–47)	***0.001***
Male sex	23 (55%)	38 (56%)	32 (55%)	0.909
Symptoms and signs				
• Cough	17 (40%	44 (64%)	11 (19%)	***0.014***
• Vomiting	17 (40%)	27 (39%)	31 (53%)	0.936
• Headache	19 (45%)	15 (22%)	30 (52%)	0.175
• Diarrhea	16 (38%)	19 (28%)	31 (53%)	0.268
• Myalgia	13 (31%)	11 (16%)	36 (62%)	***0.013***
• Rash	3 (7%)	5 (7%)	10 (17%)	0.967
• Lymphadenopathy	5 (12%)	0	0	***0.001***
• Eschar	3 (7%)	0	0	***0.001***
Biomarkers				
• White cell counts	7.5 (IQR 4.6–11.2)	11.2 (IQR 18.7)	2.8 (IQR 2–5)	***0.001***
• Platelets	173 (IQR 80–239)	212 (IQR 125–326)	61 (IQR 29–113)	***0.001***
• Procalcitonin	0.44 (IQR 0.15–1.2)	1.9 (IQR 0.29–31)	0.29 (IQR 0.15–0.65)	***0.018***
• C-reactive protein	49 (IQR 7–96)	103 (IQR 42–218)	12 (IQR 3–18)	***0.001***
Sepsis severity and outcome				
• Severe sepsis by qSOFA (≥2) ^†^	9 (21%)	23 (34%)	0 (0%)	***0.001***
• Deaths	4 (10%)	16 (24%)	0(0%)	***0.001***

Data are n (%) unless otherwise indicated. ^†^ Quick sequential organ failure assessment (qSOFA); 1 point each for systolic blood pressure < 100 mmHg, respiratory rate > 22/min, and altered mental status with Glasgow coma scale < 15. * All rickettsial infections combine all patients with monoinfection with scrub, murine, or spotted fever rickettsial typhus.

## Data Availability

The data presented in this study are available on request from the corresponding author. The data are not publicly available due to ethical reason.
