# Correction: Yuhana et al. Rickettsial Infections Are Neglected Causes of Acute Febrile Illness in Teluk Intan, Peninsular Malaysia. *Trop. Med. Infect. Dis*. 2022, *7*, 77

**DOI:** 10.3390/tropicalmed7070134

**Published:** 2022-07-13

**Authors:** Muhamad Yazli Yuhana, Borimas Hanboonkunupakarn, Ampai Tanganuchitcharnchai, Pimpan Sujariyakul, Piengchan Sonthayanon, Kesinee Chotivanich, Sasithon Pukrittayakamee, Stuart D. Blacksell, Daniel H. Paris

**Affiliations:** 1 Department of Clinical Tropical Medicine, Faculty of Tropical Medicine, Mahidol University, Bangkok 10400, Thailand; aleeyuhana@hotmail.com (M.Y.Y.); nok@tropmedres.ac (K.C.); yon@tropmedres.ac (S.P.); 2 Department of Infectious Diseases and Tropical Medicine, School of Medicine, Universiti Teknologi MARA (UiTM), Sg Buloh Campus, Sungai Buloh 40600, Selangor, Malaysia; 3 Mahidol-Oxford Tropical Medicine Research Unit, Faculty of Tropical Medicine, Mahidol University, Bangkok 10400, Thailand; ampai@tropmedres.ac (A.T.); ps.pimpan@hotmail.com (P.S.); stuart@tropmedres.ac (S.D.B.); 4 Department of Molecular Tropical Medicine and Genetics, Faculty of Tropical Medicine, Mahidol University, Bangkok 10400, Thailand; piengchan@tropmedres.ac; 5 Centre for Tropical Medicine, Nuffield Department of Clinical Medicine, Churchill Hospital, Oxford OX3 7FZ, UK; 6 Faculty of Medicine, University of Basel, 4003 Basel, Switzerland; daniel.paris@unibas.ch; 7 Department of Medicine, Swiss Tropical and Public Health Institute, 4123 Allschwil, Switzerland

The authors wish to make the following correction to this paper [1]. In Section 2.1, an error appears in ethic approval number, in which the number MUTM 2016-013-1 incorrectly instead of the correct number 2016-028-01. In the “Institutional Review Board Statement” part of the paper, the ethic approval number should be 2016-028-01 as well. The corrected text appears below.

Full ethical clearance was obtained from the Malaysian National Medical Research Register Ethical Committee (NMRR-15-756-25320) and the Ethic Committee of the Faculty of Tropical Medicine, Mahidol University (MUTM 2016-028-01). All patients enrolled provided written informed consent prior to collection of sample specimens.

**Institutional Review Board Statement**: The study was conducted in accordance with the Declaration of Helsinki and approved by the Malaysian National Medical Research Register Ethical Committee (NMRR-15-756-25320) and the Ethic Committee of the Faculty of Tropical Medicine, Mahidol University (MUTM 2016-028-01).

The authors apologize for any inconvenience caused and state that the scientific conclusions are unaffected. This correction was approved by the Academic Editor. The original publication has also been updated.
